# Supplementary material for: Multiplexed mRNA assembly into ribonucleoprotein particles plays an operon-like role in the control of yeast cell physiology
Source: eLife. 2021 May 4;10:e66050. doi: 10.7554/eLife.66050 (PMC8137142; doi:10.7554/eLife.66050)
Supplement: Supplementary file 2. — In this table, we include the strain name, genotype, and source of origin. [file elife-66050-supp2.docx]

**Supplementary File 2. Yeast strains used in this study**

| **Name** | **Genotype** | **Source** |
| --- | --- | --- |
| BY4741 | *MAT*a *his3Δ1 leu2Δ0 met15Δ0 ura3Δ0* | Euroscarf |
| BY4742 | *MAT*α *his3Δ1 leu2Δ0 lys2Δ0 ura3Δ0* | Euroscarf |
| *ABP1_INT_*  (BY4742) | *MAT*α *his3Δ1 leu2Δ0 lys2Δ0 ura3Δ0 ABP1::loxP::MS2L::ABP1^3’-UTR^* | J. Gerst |
| *ASH1_INT_*  (BY4742) | *MAT*α *his3Δ1 leu2Δ0 lys2Δ0 ura3Δ0 ASH1::loxP::MS2L::ASH1^3’-UTR^* | J. Gerst |
| *ATG8_INT_*  (BY4742) | *MAT*α *his3Δ1 leu2Δ0 lys2Δ0 ura3Δ0 ATG8::loxP::MS2L::ATG8^3’-UTR^* | J. Gerst |
| *EXO70_INT_*  (BY4742) | *MAT*α *his3Δ1 leu2Δ0 lys2Δ0 ura3Δ0 EXO70::loxP::MS2L::EXO70^3’-UTR^* | J. Gerst |
| *MFα1_INT_*  (BY4742) | *MAT*α *his3Δ1 leu2Δ0 lys2Δ0 ura3Δ0 MFα1::loxP::MS2L::MFα1 ^3’-UTR^* | This study |
| *MFα2_INT_*  (BY4742) | *MAT*α *his3Δ1 leu2Δ0 lys2Δ0 ura3Δ0 MFα2::loxP::MS2L::MFα2^3’-UTR^* | This study |
| *MYO2_INT_*  (BY4742) | *MAT*α *his3Δ1 leu2Δ0 lys2Δ0 ura3Δ0 MYO2::loxP::MS2L::MYO2^3’-UTR^* | J. Gerst |
| *MYO4_INT_*  (BY4742) | *MAT*α *his3Δ1 leu2Δ0 lys2Δ0 ura3Δ0 MYO4::loxP::MS2L::MYO4^3’-UTR^* | J. Gerst |
| *OM45_INT_*  (BY4742) | *MAT*α *his3Δ1 leu2Δ0 lys2Δ0 ura3Δ0 OM45::loxP::MS2L::OM45^3’-UTR^* | J. Gerst |
| *OXA1_INT_*  (BY4742) | *MAT*α *his3Δ1 leu2Δ0 lys2Δ0 ura3Δ0 OXA1::loxP::MS2L::OXA1^3’-UTR^* | J. Gerst |
| *PEX14_INT_*  (BY4742) | *MAT*α *his3Δ1 leu2Δ0 lys2Δ0 ura3Δ0 PEX14::loxP::MS2L::PEX14^3’-UTR^* | J. Gerst |
| *SAG1_INT_*  (BY4742) | *MAT*α *his3Δ1 leu2Δ0 lys2Δ0 ura3Δ0 SAG1::loxP::MS2L::SAG1^3’-UTR^* | This study |
| *SEC4_INT_*  (BY4742) | *MAT*α *his3Δ1 leu2Δ0 lys2Δ0 ura3Δ0 SEC4::loxP::MS2L::SEC4^3’-UTR^* | J. Gerst |
| *SRO7_INT_*  (BY4742) | *MAT*α *his3Δ1 leu2Δ0 lys2Δ0 ura3Δ0 SRO7::loxP::MS2L::SRO7^3’-UTR^* | J. Gerst |
| *STE3_INT_*  (BY4742) | *MAT*α *his3Δ1 leu2Δ0 lys2Δ0 ura3Δ0 STE3::loxP::MS2L::STE3^3’-UTR^* | This study |
| *AGA1_INT_*  (BY4741) | *MAT*a *his3Δ1 leu2Δ0 met15Δ0 ura3Δ0 AGA1::loxP::MS2L::AGA1^3’-UTR^* | This study |
| *AGA2_INT_*  (BY4741) | *MAT*a *his3Δ1 leu2Δ0 met15Δ0 ura3Δ0 AGA2::loxP::MS2L::AGA2^3’-UTR^* | This study |
| *ASH1_INT_*  (BY4741) | *MAT*a *his3Δ1 leu2Δ0 met15Δ0 ura3Δ0 ASH1::loxP::MS2L::ASH1^3’-UTR^* | J. Gerst |
| *MFA1_INT_*  (BY4741) | *MAT*a *his3Δ1 leu2Δ0 met15Δ0 ura3Δ0 MFA1::loxP::MS2L::MFA1^3’-UTR^* | This study |
| *MFA2_INT_*  (BY4741) | *MAT*a *his3Δ1 leu2Δ0 met15Δ0 ura3Δ0 MFA2::loxP::MS2L::MFA2^3’-UTR^* | This study |
| *MFA1_INT_ 3’UTRΔ*  (BY4741) | *MAT*a *his3Δ1 leu2Δ0 met15Δ0 ura3Δ0 MFA1::loxP::MS2L::MFA1^3’-UTRΔ::HIS3^* | This study |
| *MFA2_INT_ 3’UTRΔ*  (BY4741) | *MAT*a *his3Δ1 leu2Δ0 met15Δ0 ura3Δ0 MFA2::loxP::MS2L::MFA2^3’-UTRΔ::HIS3^* | This study |
| *STE2_INT_*  (BY4741) | *MAT*a *his3Δ1 leu2Δ0 met15Δ0 ura3Δ0 STE2::loxP::MS2L:: STE2^3’-UTR^* | This study |
| *STE2_INT_ AGA2_INT_*  (BY4741) | *MAT*a *his3Δ1 leu2Δ0 met15Δ0 ura3Δ0 STE2::loxP::MS2L:: STE2^3’-UTR^ AGA2::loxP::PP7L:: AGA2^3’-UTR^* | This study |
| *STE2_INT_ 3’UTRΔ*  (BY4741) | *MAT*a *his3Δ1 leu2Δ0 met15Δ0 ura3Δ0 STE2::loxP::MS2L::STE2^3’-UTRΔ::HIS3^* | This study |
| *STE2_INT_ 3’UTR*  *DHH1-m*Cherry  (BY4741) | *MAT*a *his3Δ1 leu2Δ0 met15Δ0 ura3Δ0 DHH1::mCherry::natMx STE2::loxP::MS2L::STE2^3’-UTR^* | This study |
| *STE5_INT_ bar1Δ*  (BY4741) | *MAT*a *his3Δ1 leu2Δ0 met15Δ0 ura3Δ0 STE5::loxP::MS2L::STE5^3’-UTR^ bar1Δ::natMx* | This study |
| *STE5_INT_ FUS3_INT_* (BY4741) | *MAT*a *his3Δ1 leu2Δ0 met15Δ0 ura3Δ0 STE5::loxP::MS2L::STE5^3’-UTR^ FUS3::loxP::PP7L:: FUS3^3’-UTR^*  *bar1Δ::natMx* | This study |
| *UGO1_INT_* (BY4741) | *MAT*a *his3Δ1 leu2Δ0 met15Δ0 ura3Δ0 UGO1::loxP::MS2L::UGO1^3’-UTR^* | This study |
| *UGO1_INT_ TOM6 _INT_* (BY4741) | *MAT*a *his3Δ1 leu2Δ0 met15Δ0 ura3Δ0 UGO1::loxP::MS2L::UGO1^3’-UTR^ TOM6::loxP::PP7L::TOM6^3’-UTR^* | This study |
| *bar1Δ*  (BY4741) | *MAT*a *his3Δ1 leu2Δ0 met15Δ0 ura3Δ0 bar1Δ:: hphMx* | This study |
| *dhh1Δ* (BY4741) | *MATa his3Δ1 leu2Δ0 met15Δ0 ura3Δ0 dhh1Δ::natMx* | This study |
| *dhh1Δ pat1Δ*  (BY4741) | *MATa his3Δ1 leu2Δ0 met15Δ0 ura3Δ0 dhh1Δ::natMx*  *pat1Δ::hphMx* | This study |
| *hat1Δ*  (BY4741) | *MATa his3Δ1 leu2Δ0 met15Δ0 ura3Δ0 sas2Δ::natMx* | This study |
| *hat2Δ*  (BY4741) | *MATa his3Δ1 leu2Δ0 met15Δ0 ura3Δ0 htb1Δ::natMx* | This study |
| *hhf1Δ*  (BY4741) | *MAT*a *his3Δ1 leu2Δ0 met15Δ0 ura3Δ0 hhf1Δ::natMx* | This study |
| *hhf2Δ*  (BY4741) | *MAT*a *his3Δ1 leu2Δ0 met15Δ0 ura3Δ0 hhf2Δ::natMx* | This study |
| *HA-AID-HHF1 hhf2Δ*  (BY4741) | *MAT*a *his3Δ1 leu2Δ0 met15Δ0 ura3Δ0 HHF1::HA-AID-hhf1*  *hhf2Δ:: prTIR1-TEF3* | This study |
| *hhf1^K-R^*  (BY4741) | *MATa his3Δ1 leu2Δ0 met15Δ0 ura3Δ0 hhf1^K-R^* | This study |
| *hhf1Δ*  *scp160Δ*  (BY4741) | *MATa his3Δ1 leu2Δ0 met15Δ0 ura3Δ0 hhf1Δ::natMx scp160Δ::hphMx* | This study |
| *hht1Δ*  (BY4741) | *MAT*a *his3Δ1 leu2Δ0 met15Δ0 ura3Δ0 hht1Δ::natMx* | This study |
| *hht2Δ*  (BY4741) | *MAT*a *his3Δ1 leu2Δ0 met15Δ0 ura3Δ0 hht2Δ::natMx* | This study |
| *hta1Δ*  (BY4741) | *MAT*a *his3Δ1 leu2Δ0 met15Δ0 ura3Δ0 hta1Δ::natMx* | This study |
| *hta2Δ*  (BY4741) | *MAT*a *his3Δ1 leu2Δ0 met15Δ0 ura3Δ0 hta2Δ::natMx* | This study |
| *htb1Δ*  (BY4741) | *MAT*a *his3Δ1 leu2Δ0 met15Δ0 ura3Δ0 htb1Δ::natMx* | This study |
| *htb2Δ*  (BY4741) | *MAT*a *his3Δ1 leu2Δ0 met15Δ0 ura3Δ0 htb2Δ::natMx* | This study |
| *pat1Δ* (BY4741) | *MATa his3Δ1 leu2Δ0 met15Δ0 ura3Δ0 pat1Δ::natMx* | This study |
| *sas2Δ*  (BY4741) | *MATa his3Δ1 leu2Δ0 met15Δ0 ura3Δ0 sas2Δ::natMx* | This study |
| *scp160Δ*  BY4742 | *MATα his3Δ1 leu2Δ0 lys2Δ0 ura3Δ0 scp160Δ::natMx* | This study |
| GFPlacI tetR-3xCFP  (W303) | *MATa leu2-3,112 trp1-1 can1-100 ura3-1 ade2-1*::*tetR*-*X3CFP*::*hphMx his3-11,15*::*GFPlacI::HIS3* | Dovrat *et al.* 2018 |
| GFPlacI tetR- tdtomato  (W303) | *MATa leu2-3,112 trp1-1 can1-100 ura3-1 ade2-1*::*tetR-td* *tomato*::*kanMx his3-11,15::GFPlacI::HIS3* | This study |
| GFPLacI tetR- tdtomato *AGA2* 224×tetR and *STE2* 256×lacO  (W303) | *MATa leu2-3,112 trp1-1 can1-100 ura3-1 ade2-1*::*tetR-tdtomato*::*kanMx his3-11,15*::*GFPlacI::HIS3 AGA2::224×tetR::LEU2::AGA2^3’-UTR^*  *STE2::256×lacO::TRP1::STE2 ^3’-UTR^* | This study |
| GFPlacI tetR- tdtomato *AGA2* 224×TetR and *ASH1* 256×lacO  (W303) | *MATa leu2-3,112 trp1-1 can1-100 ura3-1 ade2-1*::*tetR-td tomato::kanMx his3-11,15::GFPlacI::HIS3 AGA2::224×TetR LEU2::*AGA2^3’-UTR^ *ASH1*::*256×LacO TRP1::ASH1 ^3’-UTR^* | This study |
| GFPLacI tetR- tdtomato *AGA2* 224×tetR and *STE2* 256×lacO *hhf1Δ*  (W303) | *MATa leu2-3,112 trp1-1 can1-100 ura3-1 ade2-1*::*tetR-tdtomato*::*kanMx his3-11,15*::*GFPlacI::HIS3 AGA2::224×tetR::LEU2::AGA2^3’-UTR^*  *STE2::256×lacO::TRP1::STE2 ^3’-UTR^* *hhf11Δ::natMx* | This study |
