# Supplementary material for: Multiplexed mRNA assembly into ribonucleoprotein particles plays an operon-like role in the control of yeast cell physiology
Source: eLife. 2021 May 4;10:e66050. doi: 10.7554/eLife.66050 (PMC8137142; doi:10.7554/eLife.66050)
Supplement: Supplementary file 3. — In this table, we include the plasmid name, gene expressed, vector backbone used, copy number of the plasmid, selection marker, and source of origin. [file elife-66050-supp3.docx]

**Supplementary Table 3. Plasmids used in this study**

| **Plasmid name** | **Gene expressed** | **Vector** | **Copy**  **number** | **Selection**  **marker** | **Source** |
| --- | --- | --- | --- | --- | --- |
| pMS2-CP-GFPx3 | *MET25p-MS2-CP-GFP(×3)* | pCP-GFP  (pUG23 base) | *CEN* | *HIS3* | J. Gerst |
| pMS2-CP-GFPx3 | *MET25p-MS2-CP-GFP(×3)* | pCP-GFP  (pUG23 base) | *CEN* | *URA3* | J. Gerst |
| pAD54-Hhf1 | *HA-HHF1* | pAD54 | 2µ | *LEU2* | This Study |
| prs426-Cas9-sgDNA-natMx | Cas9  sgRNA against *natMx* marker | pRS426 | 2µ | *URA3* | This Study |
| prs426-Cas9-sgDNA-HO | Cas9  sgRNA against *HO* gene | pRS426 | 2µ | *URA3* | This Study |
| pmCherry-Scs2 | *mCherry-SCS2* | pAD4Δ | 2μ | LEU2 | J. Gerst |
| pLOXHIS5MS2L | *MS2L, Sphis5+* | pUG27 | *CEN* | *Sphis5+* | J. Gerst |
| pSH47 | *GALp-CRE* | - | *CEN* | *URA3* | Euroscarf |
| pAD54-Hhf1^K-R^ | *HA-HHF1^K-R^* | pAD54 | 2µ | *LEU2* | This Study |
| pAD54-Hhf1^K-Q^ | *HA-HHF1^K-Q^* | pAD54 | 2µ | *LEU2* | This Study |
| pFA6-natNT2 | - | - | - | *natMx*  *(NAT)* | Euroscarf |
| pFA6-hphMx | - | - | - | *hphMx*  *(hygromycin B)* | Euroscarf |
| pRS316-AGA2 | *AGA2* | pRS316 | *CEN* | *URA3* | This Study |
| pRS316-AGA2 27mut | *AGA2* 27 RBM mut | pRS316 | *CEN* | *URA3* | This Study |
| pRS316-AGA2 47mut | *AGA2 47* RBM mut | pRS316 | *CEN* | *URA3* | This Study |
| pSR10 | *lacOx256* array | - | - | *TRP1* | S. Gasser |
| pDtet | *tetOx224* | - | - | *LEU2* | A. Aharoni |
| pDtetR-tdTomato | *tetR-tdTomato* | - | - | *kanMx*  *(Kanamycin)* | A. Aharoni |
